# Comparison of diet quality between celiac patients and non-celiac people in East Azerbaijan-Iran

**DOI:** 10.1186/s12937-020-00561-9

**Published:** 2020-05-17

**Authors:** Zeinab Nikniaz, Reza Mahdavi, Leila Nikniaz, Zahra Akbari Namvar, Masoud Shirmohammadi, Mojgan Akhavan Sabbagh

**Affiliations:** 1 Liver and Gastrointestinal Diseases Research Center, Tabriz University of Medical Sciences, Tabriz, Iran; 2 Nutrition Research Center, Tabriz University of Medical Sciences, Tabriz, Iran; 3 Tabriz Health Services Management Research Center, Tabriz University of Medical Sciences, Tabriz, Iran; 4 Student Research Committee, Tabriz University of Medical Sciences, Tabriz, Iran

**Keywords:** Celiac disease, Diet quality, Healthy eating index

## Abstract

**Background:**

Considering the lifelong dietary restriction in celiac patients, it is important to assess the diet quality in these patients. Hence, this study aimed to investigate the diet quality in adult celiac patients and compare it with that of the non-celiac people.

**Methods:**

In the present cross-sectional study, 130 celiac patients were selected from the celiac disease (CD) registry database of East Azerbaijan province, Iran. Non-celiac people (*n* = 464) was selected from the major lifestyle promotion project conducted in the East Azerbaijan district. The dietary intake data was obtained by an 80-item semi-quantitative food frequency questionnaire. Diet quality was assessed using the healthy eating index-2015 (HEI-2015).

**Results:**

The mean total HEI score was significantly higher in the celiac group compared with the non-celiac people (*P* < 0.001) and 68.5% of non-celiac people and 17.4% of celiac patients had poor diet quality. After adjusting for confounding factors, the mean score of total HEI in adherents to gluten-free diet (GFD) was significantly higher compared with non-adherents (*P* = 0.007).

**Conclusions:**

Although the mean total HEI score was higher in celiac patients compared with the non-celiac people, about 17.5% of patients had poor diet quality and the scores of whole grains and dairy products group were very low in our population. Accordingly, it seems that educational programs should be held for the celiac patients and non-celiac people to increase their nutritional literacy and enable them to select healthy gluten-free alternatives.

## Background

Celiac disease (CD) is an autoimmune disease that presents in genetically susceptible individuals by consuming gluten-containing foods [1]. The disease is associated with different gastrointestinal and non-gastrointestinal presentations, including abdominal pain, bloating, constipation, steatorrhea, malabsorption, anemia, osteopenia and weight loss [1]. The only available treatment for CD is lifelong strict adherence to a gluten-free diet (GFD) [2]. By following this diet, the clinical symptoms are eliminated. However, considering the limitations of GFD and also the different compositions of gluten-free alternatives, there is a great concern about the adequacy and quality of this diet [3, 4].

In this regard, researchers have already drawn attention to the quality of GFD and have focused on dietary components to evaluate diet quality. However, nutritionists are emphasizing on the overall dietary quality instead of individual foods or nutrients [5]. Different indices are developed for assessing the overall dietary quality.

The HEI is a tool for evaluating the adherence to dietary guidelines and the food guide pyramid [6]. In children with CD, some studies focused on HEI [7, 8] and reported various differing results. No study has evaluated the HEI in adult celiac patients so far. Only one study assessed the diet quality through calculating the Mediterranean diet score (as a high-quality diet) and showed that the mean score of the Mediterranean diet was significantly lower in celiac patients [9].

Considering the importance of diet quality in celiac patients due to their lifelong dietary restriction and also lack of studies in adult celiac patients in this regard, the present study aimed to assess the diet quality in adult celiac patients and compare it with that of the non-celiac people.

## Materials and methods

In the present cross-sectional study, celiac patients were randomly selected from the CD registry database of East Azerbaijan province, Iran. The patients were included if they aged 20–55 years old, were registered in the CD registry database of East Azerbaijan province, and followed GFD for at least 1 year. All patients were diagnosed based on biopsy reports. The patients were excluded if they had a mental disability, did not adequately communicate with the interviewer, or had other diseases such as diabetes that affected their dietary intake.

We used the data of non-celiac participants who enrolled in the major lifestyle promotion project (LPP). LPP is a population-based study conducted in East Azerbaijan district for the evaluation of lifestyle risk factors. The detailed method of sampling and inclusion criteria have already been described [10]. For statistical analysis, 464 non-celiac participants with the age range of 20–55 years were included. The participants with the known diabetes mellitus, CD, or other diseases that affected their diet were excluded from the analysis.

### Data collection

The demographic data such as age and gender were obtained through face-to-face interviews. All anthropometric measurements were done according to the protocol of the LPP study [10]. The body weight was measured by the Seca weighing scale and height was measured by a stadiometer fixed to the wall. Body mass index (BMI) was calculated by dividing weight (kg) to height (m^2^).

The dietary intake data was obtained by a semi-quantitative food frequency questionnaire (FFQ) through face-to-face interview by an expert dietitian. HEI score was determined based on dietary intake data collected by an 80-item FFQ. This questionnaire was developed and validated for the LPP study [11]. The same questionnaire was used in celiac patients but the gluten-free items were also added in the patient’s questionnaire. Then, Modified Nutritionist IV was used for the determination of food energy and macro- and micronutrient content. This software has the composition of Iranian food and gluten-free foods. Moreover, for calculating diet quality, all food consumption data were converted into serving size equivalents based on the US Department of Agriculture (USDA) databases. The macro- and micronutrient data were used for HEI scores calculation.

Overall diet quality was assessed using HEI-2015. The HEI-2015 total score and components scores were calculated based on the 2015–2020 Dietary Guidelines for Americans (DGAs) and the USDA standards for HEI-2015 [12]. For determining HEI total score, the score of the 13 components (including total fruit, whole fruit, dark green vegetables and legumes, whole grains, dairy products, total protein foods, sea foods and plant proteins, fatty acid ratio, refined grain, sodium, added sugars, and saturated fat) were summed and the participants were stratified into “poor” (a HEI score of 50 or less), “needs improvement” (a HEI score of between 51 and 80), and “good” (a HEI score of 81 or more) diet quality categories [13].

### Assessing adherence to the CD

Adherence to the GFD in CD participants was determined by an anti-tissue transglutaminase (tTG) serology test using AESKULISA® tTG new generation kit. According to Kit instruction, the patients with values greater than 10 IU/ml were considered as non-adherents.

### Statistical analysis

For statistical analyses, SPSS V.22 was used. Skewness and kurtosis data were used to verify the normality assumption. The independent t-test, chi-square, and Fisher exact tests were used for the comparison of dietary intake and general and anthropometric characteristics between groups. The one-way ANCOVA was used for comparing the HEI between groups by adjusting to confounding factors such as age, sex, BMI, and energy intake. A significance level of 0.05 was used.

## Results

**Fig. 1:**
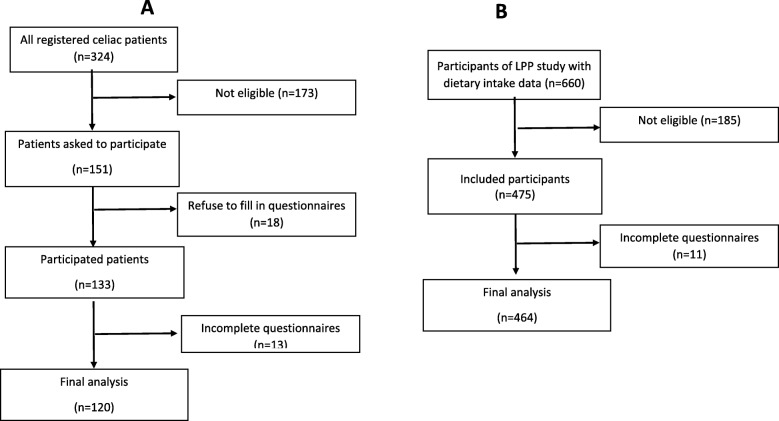
Study enrolment flowchart: **a** celiac patients enrolment **b** healthy population enrolment

As shown in Fig. 1, in the celiac group, 10 participants were excluded from the final analysis due to incomplete questionnaires and final analysis was done on 120 patients. The mean age of participants and the mean disease duration was 36.7 ± 8.6 and 6.4 ± 8.1 years, respectively. The characteristics of participants in CD and non-celiac people groups are presented in Table 1. The mean age of celiac patients was similar to that of the non-celiac people (*P* = 0.2). There were significant differences between groups regarding anthropometric characteristics (*P* < 0.05). Significantly more participants in the non-celiac people group were overweight/obese compared with celiac patients and the mean energy intake in celiac patients was significantly lower than that of non-celiac people (*P* < 0.001). The comparison of the mean BMI level between GFD adherents (23.8 ± 3.6) and non-adherents (23.5 ± 3.8) revealed that there was no significant difference between the groups (*P* = 0.6).

**Table 1:** demographic and anthropometric characteristics of celiac patients and healthy population

Variables	Celiac patients (*n* = 120)	Healthy people (*n* = 464)	*P*-value*
**Age (years)**	36.7 ± 8.6	37.9 ± 9.5	0.2
**Sex (M:F)**	37 (32.1)/78 (67.8)	206 (44.3)/258 (55.6)	0.02
**Weight (kg)**	63.2 ± 10.9	73.0 ± 13.3	< 0.001
**Height (cm)**	163.0 ± 9.8	164.4 ± 12.9	0.2
**Waist circumference**	84.0 ± 12.5	90.9 ± 13.4	< 0.001
**Hip circumference**	98.4 ± 10.0	103.0 ± 11.6	< 0.001
**BMI (kg/m**^**2**^**)**	23.8 ± 3.7	27.4 ± 9.2	< 0.001
**Underweight**	8 (7.00)	14 (3)	0.2
**Normal weight**	66 (57.4)	142 (30.6)	< 0.001
**Overweight/obese**	41 (35.7)	284 (61.2)	< 0.001
**Energy intake (Kcal/day)**	2324 ± 961.4	3445 ± 1459	< 0.001

*BMI* body mass index

*P-value of independent t-test

The HEI components and total scores are presented in Table 2. The patients with CD had significantly lower scores in terms of whole grains (*P* < 0.001), sea foods, and plant proteins (*P* < 0.001). The mean total HEI score was significantly higher in the celiac group compared with the non-celiac group (*P* < 0.001). According to the HEI criteria, in the non-celiac group, 68.5% of participants had a poor diet quality and 31.5% of them had a diet that needs improvement. However, in the celiac group, 17.4% of patients had a poor diet quality and 82.6% of them had a diet that needs improvement. Additionally, we compared the total HEI score and sub-scores in adherents and non-adherents to GFD. The results of one-way ANCOVA showed that after adjusting for age, sex, BMI, energy intake, disease duration, and treatment duration, the mean score of total HEI in adherents to GFD (59.64 ± 7.56) was significantly higher compared with non-adherents (56.2 ± 7.7) (*P* = 0.007). Also, there was no significant difference between groups regarding HEI components except for the refined grains score that was significantly higher in GFD adherents compared with non-adherents (6.1 ± 4.5 Vs. 4.8 ± 4.9; *P* = 0.005).

**Table 2:** comparison of the HEI components and total scores in celiac group and healthy people

Healthy eating index scores	Max score	Standard for Max Score	Celiac patients			Healthy people	*P*-value *	*P*-value **
			Total	Adherents	Non-adherents			
**Total fruit**	5	≥0.8 cup eq/1000 kcal	4.0 ± 1.4	4.1 ± 1.4	3.8 ± 1.6	1.9 ± 1.3	0.4	< 0.001
**Whole fruit**	5	≥0.4 cup eq/1000 kcal	4.4 ± 1.20	4.5 ± 1.2	4.3 ± 1.3	2.9 ± 1.5	0.3	0.005
**Total vegetables**	5	≥1.1 cup eq/1000 kcal	4.7 ± 0.6	4.6 ± 0.7	4.7 ± 0.5	4.3 ± 0.9	0.6	0.06
**Dark green vegetables and legumes**	5	≥0.2 cup eq/1000 kcal	4.9 ± 0.1	4.9 ± 0.1	4.9 ± 0.1	4.8 ± 0.6	0.9	0.6
**Whole grains**	10	≥1.5 oz. eq/1000 kcals	0.08 ± 0.3	0.08 ± 0.4	0.05 ± 0.2	2.0 ± 2.7	0.5	< 0.001
**Dairies**	10	≥1.3 cup eq/1000 kcals	4.7 ± 3.2	5.4 ± 3.2	4.3 ± 3.2	4.4 ± 2.4	0.1	0.8
**Total protein foods**	5	≥2.5 oz. eq/1000 kcals	4.7 ± 0.8	4.6 ± 0.9	4.8 ± 0.5	2.4 ± 1.0	0.2	< 0.001
**Sea foods and plant proteins**	5	≥0.8 oz. eq/1000 kcals	0.1 ± 0.6	0.06 ± 0.3	0.02 ± 0.1	2.8 ± 1.3	0.2	< 0.001
**Fatty acid ratio**	10	(PUFAs + MUFAs)/SFAs ≥2.5	5.4 ± 3.8	5.6 ± 3.8	5.5 ± 3.6	2.2 ± 2.1	0.7	< 0.001
**Refined grain**	10	≤1.8 oz. eq/1000 kcals	5.9 ± 4.7	6.8 ± 4.5	4.8 ± 4.9	3.2 ± 3.8	0.005	< 0.001
**Sodium**	10	≤1.1 g/1000 kcals	1.2 ± 2.8	1.4 ± 3.0	0.8 ± 2.3	0.1 ± 1.0	0.5	< 0.001
**Added sugars**	10	≤6.5% of energy intake	9.6 ± 1.1	9.7 ± 1.2	9.6 ± 1.0	9.2 ± 2.1	0.6	0.06
**Saturated fats**	10	≤8% of energy intake	7.6 ± 2.6	7.5 ± 2.4	8.1 ± 2.7	9.4 ± 2.1	0.4	< 0.001
**Total score**	100	–	57.8 ± 7.9	59.6 ± 7.5	56.2 ± 7.7	43.1 ± 14.1	0.007	< 0.001

*P-value of ANCOVA comparing adherent and non-adherent celiac patients adjusted for age, sex, BMI, energy intake; disease duration and treatment duration

**P-value of ANCOVA comparing celiac disease and healthy population adjusted for age, sex, BMI, energy intake

## Discussion

There is little evidence regarding the diet quality of adult celiac patients and whether the diet quality of these patients is different from that of the non-celiac people. In the present study, we showed that the mean total HEI score in patients with CD was significantly higher than that of the general population. Moreover, 17.4% of patients with CD had a poor diet quality and the remaining had a diet that needs improvement. However, these values in the non-celiac people were 68.5 and 31.5%, respectively. As far as the researchers investigated, this is the first study to assess the diet quality using HEI-2015 in the adult celiac patients. In a study in children with CD, Liu et al. showed that about 78% of children had a diet that needs improvement and none of them had a poor diet quality [8]. In another study in children, Mager et al. compared the diet quality in children with CD and control group (with other gastrointestinal disorders) and showed that the HEI score of celiac patients was significantly higher than that of the control group [7]. However, Alzaben et al. reported no differences in HEI score between children with CD and healthy controls [14]. In the adult celiac patients, the observed higher score of HEI in celiac patients compared with the non-celiac people could be related to the higher nutritional knowledge of celiac patients due to the regular dietary counseling and also paying more attention to the diet. In addition, as they can’t eat most packaged or processed foods, they had healthier diet.

Although the total score of HEI in celiac patients was higher than that of the non-celiac people, about 17.4% of patients had a poor diet quality. This may be due to the unavailability of commercial gluten-free alternatives or lack of knowledge about adding healthy foods to GFD. For example in the present study, the lowest score in the CD group was related to the whole grains group. Generally, whole grains are mostly consumed as bread, but there is no available commercial gluten-free alternative for whole bread in Iran. In addition, they are not as appealing to some patients and are more expensive.

In the present study, there was no statistically significant difference in score of the dairy product consumption between celiac patients and the control group. In their study, Babio et al. also reported no significant differences between children with CD and healthy controls regarding the adherence to dairy products recommendations [15]. Some previous studies reported that the intake of dairy products in Iran was lower than the recommended number of dairy servings [12, 16]. This may be due to lactose intolerance. So, they should be educated to choose low lactose dairy foods such as cheese or alternative sources of calcium.

Moreover, celiac patients significantly scored lower in plant protein and legumes group. This finding could be partially explained by this fact that non-digestible carbohydrates found in pulses can cause bloating [17]. Considering the high prevalence of bloating in celiac patients [18], these patients tended to decrease the consumption of this food group. However, this group is a good source of protein, fiber, and micronutrients [19]. Thus, patients should be educated about the appropriate approaches to decrease the flatulence causing factors of legumes.

The score of sodium consumption in celiac patients was significantly higher than that of the non-celiac people; this may be related to the low content of sodium in GFD. Earlier studies also reported low consumption of sodium in celiac patients compared with healthy controls [20, 21]. However, in both groups, the consumption of sodium was much higher than the recommendations. A previous study in Iran also reported high consumption of salt in the Iranian adult population [22].

In the present study, we showed that compared with non-adherents to GFD, the patients with strict compliance had also better adherence to the recommendations regarding refined carbohydrate consumption. However, in a study conducted in children with CD, Alzaben et al. reported the higher consumption of high glycemic index (GI) and glycemic load (GL) foods in adherent celiac patients [14]. In addition, the total HEI score was significantly higher in adherent patients compared with non-adherents. However, this finding does not support the results of a previous study in which Mager et al. reported no association between diet quality (assessed by dietary pattern method and DASH score) and adherence to GFD [7]. The observed controversy between the results of these studies may be due to the difference in included population, the method of defining non-adherence, and the method of evaluation of the diet quality.

The findings of this study should be interpreted cautiously for a few reasons. We used FFQ for assessing dietary intake and its limitations such as recall bias may have influenced some of the findings. However, the FFQ was validated in the general population of East Azerbaijan and modified to assess the dietary habits of individuals with CD and fulfilled by a trained nutritionist. Besides, we used HEI for assessing diet quality. This index does not address the complex factors such as behavioral and psychosocial aspects that are important to meal patterning. Another limitation of the present study was the inclusion of a limited number of celiac patients. However, these patients were selected randomly from a computerized database of > 300 biopsy-confirmed cases. Furthermore, we used the data of the previous study that assessed the dietary intakes in the population of East Azerbaijan as a non-celiac population instead of a concurrently collected control group. Although we excluded the patients with diabetes and apparently celiac patients, some of the CD cases may remain undiagnosed [23]; accordingly, we cannot be confident that all participants in the non-celiac population group are free of disease. Besides, we used anti-tissue transglutaminase (tTG) serology test for categorizing patients as adherents and non-adherants, however, a normal tTG does not guarantee adherence.

## Conclusion

In conclusion, the results of the present study showed that although the mean total score of HEI was higher in celiac patients compared with the non-celiac population, about 17.5% of patients had a poor diet quality and the scores of whole grains and dairy products group and sodium consumption were very low in our sample. Thus, from a practical point of view, educational programs should be held for the celiac patients and non-celiac people to increase their nutritional literacy and enable them to select healthy gluten-free alternatives. From the research point of view, it is suggested that future studies use a more valid tool for evaluating dietary intake in a large population of celiac patients and compare them with the concurrently collected control group for more precise results.

## Data Availability

The datasets supporting the conclusions of this research are included within the article.
